# Natural chunk-and-pass language processing: Just another joint source-channel coding model?

**DOI:** 10.1080/19420889.2018.1445899

**Published:** 2018-04-27

**Authors:** Kevin B. Clark

**Affiliations:** a Research and Development Service, Veterans Affairs Greater Los Angeles Healthcare System, Los Angeles, CA, USA; b NeuroLex Laboratories, Inc., Atlanta, GA, USA; c Oregon Bioscience Incubator, Oregon Translational Research and Development Institute, Portland, OR, USA; d Expert Network, Penn Center for Innovation, University of Pennsylvania, Philadelphia, PA, USA

**Keywords:** fault-tolerance and correction, hierarchical language representation, language coding and decoding, language compression, multilevel lossy code-transmission rate and security, natural language acquisition and pattern duality, shannon's noisy channel coding and separation theorems

## Abstract

A recent theoretical treatment by Christiansen and Chater attempts to address fundamental challenges significant to language processing and evolution with one major operational constraint called the “Now-or-Never” bottleneck. The authors' “Chunk-and-Pass” processing putatively mitigates the severe multilevel Now-or-Never bottleneck via fast linguistic coding and compression, hierarchical language representation and pattern duality, and incrementally learned item-based predictions useful for grammaticalization over wide spacetime scales. Despite being a promising explanation of language processes, structure, and development, the Chunk-and-Pass model manages the Now-or-Never constraint with seeming reliance on optimal joint source-channel coding, a set of computational attributes for natural and artificial speech based on Shannon's noisy channel theorems. Restating the Now-or-Never bottleneck with information-theoretic source-channel capacity limitations stresses tradeoffs inherent in the authors' model involving multilevel lossy code-transmission rate and security. Such attributes render evolvable associative networks capable of Chunk-and-Pass speech acquisition, recognition, generation, and adaptation, suggesting Chunk-and-Pass processing represents a special case of joint source-channel coding.

Christiansen and Chater [1] conceive and characterize their hypothetical “Chunk-and-Pass” language-processing model in a welcome attempt to resolve enduring, if uncontroversial, practical and theoretical challenges in understanding natural language coding/decoding, compression, and fault tolerance/correction. The authors unite these well-known fundamental challenges, significant to both language processing and evolution [e.g., 2–7], into one major operational constraint which they call the “Now-or-Never” bottleneck – the very transient, error-prone persistence of perceived and generated language content that helps degrade processing quality at all levels of comprehension and production. As a perceptual-cognitive-motor mechanism, Chunk-and-Pass processing putatively mitigates the severe multilevel Now-or-Never bottleneck via fast linguistic coding and compression (i.e., “eager processing”), hierarchical language representation and pattern duality, and incrementally learned item-based predictions (i.e., “Right-First-Time” language acquisition) capable of grammaticalization over ontogenetic, sociogenetic, and even phylogenetic spacetime scales.

Yet, despite being a promising flexible explanation of language processes, structure, and development, the Chunk-and-Pass processing model manages the Now-or-Never constraint with seeming tacit reliance on optimal joint source-channel coding (JSCC), a mature set of resource-dependent computational attributes for natural and artificial speech that are derived from Shannon's noisy channel coding and separation theorems [e.g., 8–10]. The likely unintentional connection to JSCC, as the authors neglect to credit such models for their ideas, suggests certain artificial digital/analog telecommunication models display greater equivalence to natural language than Christiansen and Chater would have readers believe. Correspondence between Chunk-and-Pass processing and JSCC models thus diminishes the novelty of Christiansen and Chater's presentation, while simultaneously giving an important, albeit regrettably missed, opportunity for the authors to better shape their own arguments about the Now-or-Never bottleneck by using more complete, testable information-theoretic descriptions of language-channel capacity, code length, correlative code parsing and concatenation, code-(de)compression ratios, weighted or unequal predictive error-protection, distributed code representation, and leaned quasi-regular grammatical construction.

Identifying Chunk-and-Pass processing as a variant of JSCC grants valuable insights about Christiansen and Chater's language model with respect to the properties of noisy low-memory (or memoryless) digital, hybrid digital-analog, and near-analog JSCC models for speech coding. JSCC models, like Chunk-and-Pass processing, are limited by definite resource-allocation constraints instantiated by Shannon's noisy channel coding and separation theorems [cf. 2,9,11]. Consequently, restating the Now-or-Never bottleneck in simple terms of information-theoretic source-channel capacity limitations emphasizes those tradeoffs inherent in Christiansen and Chater's model which involve multilevel lossy code-transmission rate and security, as determined by code length, correlative code parsing (or chunking) and concatenation, mapped code-(de)compression ratios, and feedforward predictive code recovery [cf. 8–10,12–15].

Such JSCC computational attributes closely approximate information rates, fault tolerance, and scaling observed for natural language systems, including those described by Christiansen and Chater for Right-First-Time and “Just-in-Time” incremental predictive processing and for higherarchical language representation and pattern duality. Moreover, they provide a solid foundation for producing evolvable associative computational networks that exhibit ensemble coding, representational structure (e.g., weighted entropy, block parsing and sparseness, (de)compression ratios, etc.), multiresolution modulation (digital JSCC), direct source-channel mapping (hybrid digital-analog JSCC), dimension changing mapping (near analog JSCC), and unequal error-protection. These networks and their properties are capable of the kinds of Chunk-and-Process speech acquisition, recognition, generation, and adaptation which Christiansen and Charter consider necessary to mediate item-based grammaticalization and additional language changes within individuals, social groups, and neighboring generations.
